# Differential antioxidant pathways displayed by *Chlamydomonas reinhardtii* exposed to selected NSAIDs

**DOI:** 10.1038/s41598-026-57688-8

**Published:** 2026-06-15

**Authors:** Dominika Kapuścińska, Monika Hejna, Wojciech Pokora, Anna Aksmann

**Affiliations:** 1 Faculty of Biology, Department of Plant Experimental Biology and Biotechnology, University of Gdańsk, Wita Stwosza 59, Gdańsk, 80-308 Poland; 2 Faculty of Biology, Institute of Microbiology, Department of Bacterial Physiology, University of Warsaw, Ilji Miecznikowa 1, Warsaw, 02-096 Poland

**Keywords:** Microalgae, Oxidative stress, Nonsteroidal anti-inflammatory drugs, *Chlamydomonas reinhardtii*, Gene expression, Antioxidative enzymes, Biochemistry, Environmental sciences, Plant sciences

## Abstract

The chemically diverse group of nonsteroidal anti-inflammatory drugs (NSAIDs) is a significant source of aquatic pollution, inducing oxidative stress in algae. The aim of this study was to evaluate the antioxidant response of *Chlamydomonas reinhardtii* exposed to four NSAIDs differing in toxicity and structure: flufenamic acid (FFA), nabumetone (NBT), ibuprofen (IBU), and naproxen (NPX). This study demonstrated that all pharmaceuticals significantly increased H₂O₂ production, confirming redox imbalance in cells. The antioxidant defense also showed compartment- and compound-specific signatures depending on the NSAID toxicity level. Less toxic IBU and NPX induced coordinated SOD isoforms and catalase activation, while more toxic FFA and NBT triggered chloroplast-targeted H₂O₂ scavenging via APX pathways. Notably, *MSD3* transcript levels increased in all treatments, indicating its potential as an NSAID stress biomarker. IBR analysis demonstrated that antioxidant efficiency decreased with increasing NSAID toxicity. These findings demonstrate that NSAID toxicity shapes compartment- and isoform-specific antioxidant strategies in *C. reinhardtii.* We believe that future studies with a broader range of NSAIDs would enable us to investigate subcellular redox dynamics using compartment-specific ROS reporters and to identify NSAID-sensitive biomarkers for aquatic ecotoxicology monitoring.

**Supplementary Information:**

The online version contains supplementary material available at https://doi.org/10.1038/s41598-026-57688-8.

## Introduction

Pharmaceuticals are one of the most important components of modern healthcare. The effectiveness of their active compounds in the treatment of various diseases and their widespread availability contribute to their increasing consumption. However, the uncontrolled release of pharmaceutical residues and their metabolic derivatives into the environment through sewage, improper disposal, and the absence of appropriate treatment methods, as well as the insufficient legal regulations governing their remediation, poses a major challenge for worldwide ecosystems^1^.

Nonsteroidal anti-inflammatory drugs (NSAIDs), and their metabolites have been detected in the aquatic environment for many years. The range of their environmental concentrations varies depending on country-specific consumption patterns and ranges from ng/L to even several dozen of µg/L in analyzed samples^2,3^. Because NSAIDs occur in aquatic environments as diverse mixtures of parent compounds and their derivatives, their toxic effects on non-target organisms are often difficult to determine^4^. Most studies focus on animal organisms, and knowledge about the effects of NSAIDs on plant organisms, such as green microalgae, is still insufficient^3,5^.

The need for comprehensive studies on microalgae is related to their important role in aquatic ecosystems and their ability to remove many pharmaceuticals from polluted environment^6^. Therefore, to assess the potential impact of NSAIDs on microalgae communities in water bodies and to select the suitable organisms for phycoremediation, it is necessary to determine the sensitivity and resistance of green microalgae, such as *Chlamydomonas reinhardtii*, to NSAIDs with diverse chemical structures and properties, such as ibuprofen (IBU), naproxen (NPX), nabumetone (NBT), or flufenamic acid (FFA)^2^.

One of the most extensively studied eukaryotic microalgal platforms for phycoremediation is the unicellular green alga *C. reinhardtii*, and it is also recognized as a valuable biotechnological platform with applications in biofuel production, and the synthesis of high-value bioproducts due to its high proliferation rate, ease of cultivation, broad environmental adaptability, and ease of genetic and physiological manipulation^7,8^. Furthermore, its simple cellular structure is an attractive cell factory for bioenergy production. A recent study demonstrated that bicarbonate enhancement can improve bioenergy generation from *C. reinhardtii*, and acetate assimilation was identified as a promising strategy to support sustainable biofuel production. Therefore, *C. reinhardtii* has established itself as a model organism for biofuel research, including bioethanol, biodiesel, and photobiological hydrogen^8,9^. *C. reinhardtii* has also emerged as a platform for high-value bioproduct synthesis, such as the production of therapeutic antibodies, vaccines, antimicrobial peptides, and industrial enzymes^10^.

In addition to its well-established biotechnological applications, *C. reinhardtii* also serves as a valuable model organism for ecotoxicological studies, particularly in assessing the impact of emerging contaminants. The toxicity of chemicals, including NSAIDs, is typically determined based on their effective concentrations, most commonly EC50 values, which refer to the inhibition of population growth^11,12^. In our previous study on *C. reinhardtii*, NBT exhibited more than sixfold higher toxicity than FFA, with EC50 values of 5.75 mg/L and 35.47 mg/L, respectively^13^. On the other hand, ibuprofen (IBU) demonstrated relatively low toxicity to *C. reinhardtii*, with an EC50/24 of 250 mg/L^14^. Another NSAID, naproxen (NPX), exhibited a clear toxic effect on green algae, with a EC50/24 value of 29.2 mg/L for *Scenedesmus quadricauda*, and 0.7 mg/L for *Cymbella* sp^15^. At the cellular level, these compounds induce a range of physiological disruptions that additionally explain their inhibitory effects on algal growth. Further, many cellular-level disorders were detected in microalgae cells treated with NSAIDs. For instance, NPX caused changes in photosynthetic pigments content in the algal cells^15^, IBU depolarized mitochondrial and cytoplasmic membranes^14^, NBT and FFA disrupted photosynthesis^13^. However, the toxicity of NSAIDs depends on multiple factors, including the structure of the compound, exposure time, as well as the activity of oxidative enzymes, and the cell wall architecture, which varies among the microalgae species^13,16^.

Moreover, numerous studies have reported that pharmaceuticals induce oxidative stress in microalgal cells by enhancing ROS production^12,14,17,18^. ROS are standard byproducts of aerobic metabolism which are formed in almost all cellular compartments, with chloroplasts, mitochondria, and peroxisomes as primary sources. At low concentrations, ROS act as signaling molecules, however when overproduced due to biotic and abiotic stressors, they cause redox imbalance in cells^19,20^. One of the most stable forms among ROS is hydrogen peroxide, which is highly mobile, and can be synthesized in multiple cellular compartments^21^ leading to disturbances in metabolic pathways^22^. The majority of H_2_O_2_ in algal cells is produced by superoxide dismutase (SOD) while decomposing superoxide radical. In *C. reinhartdii*, the iron isoform (Fe-SOD) resides in the chloroplast, whereas the manganese isoform (Mn-SOD) is distributed in the chloroplast and mitochondria^23^. Removal of excess hydrogen peroxide generated by SOD is carried out by antioxidant enzymes such as catalase (CAT) and ascorbate peroxidase (APX)^24^. The antioxidant system not only protects cells against stress, but is also recognized as a key element responsible for cellular adaptation and increased resistance of plant organisms to environmental pollutants. Investigating the antioxidant response enables a comparison of microalgal tolerance to the tested pharmaceuticals, which may have potential applications in the biodegradation of these compounds. Moreover, considering the activity of individual enzymes and their cellular location, it is possible to indicate cellular compartments that are particularly sensitive to the applied stress factor.

In light of this, despite the growing awareness of the environmental risks associated with NSAIDs, significant gaps in current knowledge still persist. In many cases, findings obtained for individual compounds are generalized to the entire class of NSAIDs, despite the substantial structural and physicochemical diversity within this group. Therefore, comparative analyses of selected NSAIDs differing in chemical structure are essential for a more comprehensive understanding of their ecotoxicological effects. Moreover, although numerous studies report the induction of oxidative stress by NSAIDs, relatively few have addressed the intracellular sources of reactive oxygen species (ROS) or their detoxification at the molecular level. Importantly, comparative studies at this level, particularly those examining NSAIDs with distinct chemical properties, remain scarce.

Therefore, the present study aimed to compare the antioxidant response of *C. reinhardtii* to selected NSAIDs, such as flufenamic acid (FFA), nabumetone (NBT), ibuprofen (IBU) and naproxen (NPX), differing in their structure and properties. The research hypothesis assumed that each of the selected NSAIDs would cause oxidative stress in *C. reinhardtii* cells, but the mechanisms involved in minimizing its negative effects would differ. The transcriptional responses were studied in genes related to antioxidant defense, enzymatic biomarkers such as SOD, CAT, and APX were analyzed, and hydrogen peroxide produced by microalgae was determined.

## Results

### Population density and hydrogen peroxide production by the cells

**Fig. 1:**
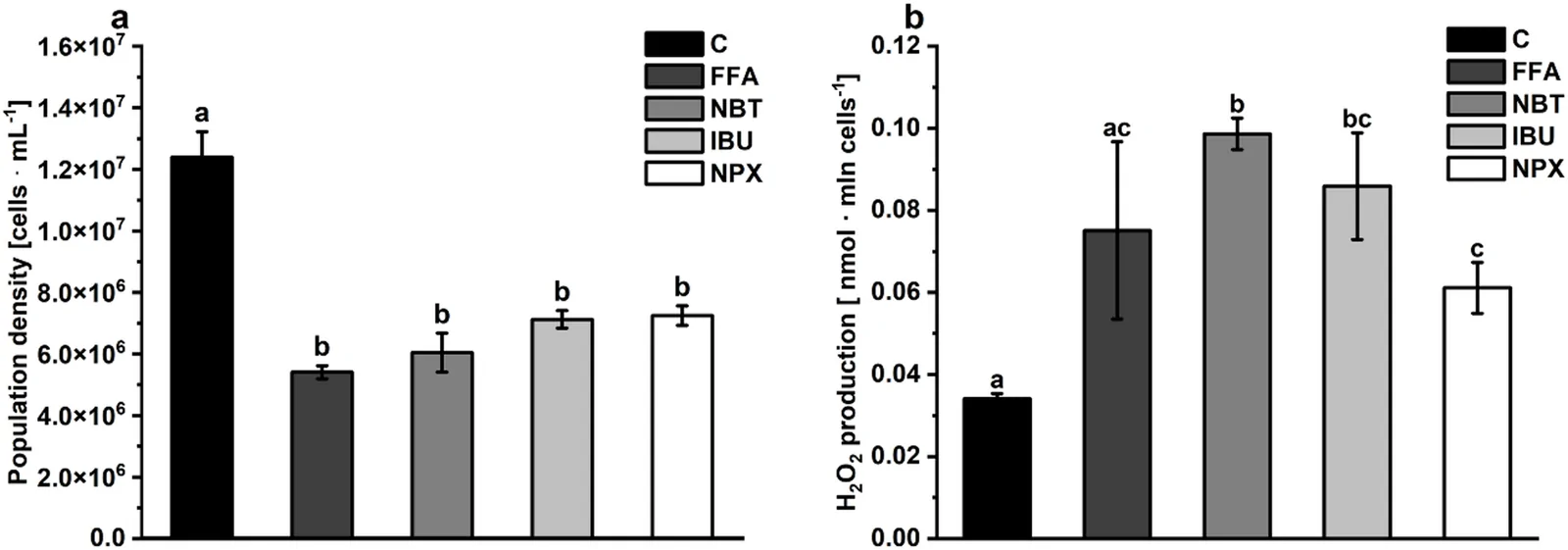
Effect of flufenamic acid (FFA), nabumetone (NBT), ibuprofen (IBU), and naproxen (NPX), applied at EC50 concentrations on population density **(a)**, and hydrogen peroxide production by *C. reinhardtii* cells **(b)**. Data are presented as averages ± SE. Different letters indicate significant differences (*p* ≤ 0.05) between groups (a- ANOVA followed by the Tukey’s HSD test, b- Kruskal-Wallis followed by the Dunn’s test).

The pharmaceuticals used in the study inhibited the growth of *C. reinhardtii* population, compared to the control, where the cell number was about 12.4 × 10^6^ cells/mL. Population density for FFA-, NBT-, IBU-, and NPX-treatment reached 44, 49, 57, and 58% of the control, respectively, and was statistically significantly lower than the control group, with no differences observed among the treated cultures (Fig. 1a). Consistently, the growth rate was significantly reduced in all treated groups from 3.13 ± 0.12 day^–1^ in the control to 2.38–2.66 day^–1^ following exposure to the tested NSAID (Tab. S2). In the control cultures, the concentration of H₂O₂ released by the cells was 0.034 nmol/million cells (Figs. 1 and 5). All NSAIDs caused a significant increase in H_2_O_2_ production by microalgae, except for FFA, where this increase did not reach statistical significance compared to the control (Figs. 1b and 5). The amount of H_2_O_2_ released by *C. reinhardtii* treated with NPX, IBU, and NBT reached 180, 252, and 290% of the control, respectively (Fig. 5).

**Fig. 2:**
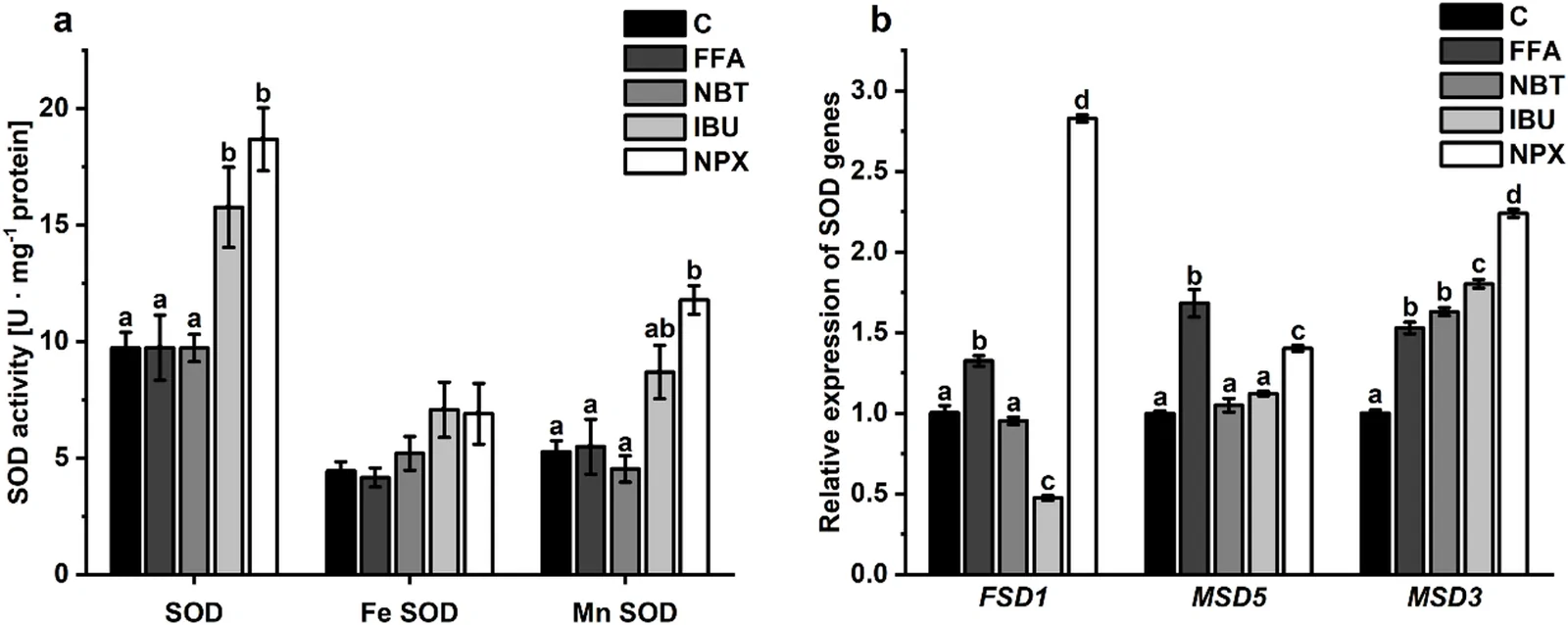
Effect of flufenamic acid (FFA), nabumetone (NBT), ibuprofen (IBU), and naproxen (NPX), applied at EC50 concentrations on activity of superoxide dismutase (SOD, **a)**, and relative level of SOD genes transcription in *C. reinhardtii* cells **(b)**. Data are presented as averages ± SE. Different letters indicate significant differences (*p* ≤ 0.05) between groups (ANOVA followed by the Tukey’s HSD test). *FSD1*: gene encoding for Fe-SOD, *MSD5*,* MSD3*: genes encoding for Mn-SODs.

### SOD activity and gene expression

Considering the high stimulation of hydrogen peroxide production by *C. reinhardtii* treated with NSAIDs, our focus was on determining the activity of superoxide dismutase, which is one of the main sources of H_2_O_2_. During PAGE analysis, two isoforms of superoxide dismutase were detected, such as Fe-SOD, known to be located in chloroplasts, and Mn-SOD, which is present in the chloroplast and mitochondria (Fig. S3)^23,25^. The activity of both SOD isoforms, and total SOD activity did not differ from the control in cells treated with FFA and NBT (Figs. 2a and 5). A significant increase in total SOD activity was observed upon exposure to IBU and NPX, reaching 15.8 U/mg and 18.7 U/mg, respectively, while the untreated cells exhibited an activity of 9.7 U/mg (Figs. 2a and 5). In cells treated with NBT, IBU, and NPX, Fe-SOD activity was enhanced, achieving 117%, 159%, and 155% of the control, respectively (Figs. 2a and 5). However, no statistically significant differences were observed. Exposure to NPX resulted in a pronounced 223% increase in Mn-SOD activity, compared to the control group. The activity of these isoforms for other NSAIDs was not statistically significant and reached 104% for FFA, 86% for NBT, and 165% for IBU (Figs. 2a and 5). To identify the transcriptional regulation of enzyme expression, we performed a more detailed analysis of the abundance of transcripts encoding superoxide dismutase. The presence of *MSD4* was not detected in any of the tested variants (data not shown). *FSD* transcript abundance was significantly increased only in FFA- and NPX-treated microalgae (132%, and 282% of control, respectively). These values for NBT and, IBU were 95% and 47% of the level determined in the control (Fig. 2b). Similarly, FFA and NPX showed a similar trend for *MSD5* transcript, where the relative gene expression was 168% and 140%, respectively, compared to the control (Figs. 2b and 5). The increase observed for cells treated with NBT and IBU was not statistically significant. In contrast to other genes, the relative expression of the *MSD3* gene significantly increased in response to all tested NSAIDs (Figs. 2b and 5). Additionally, Pearson correlation analyses showed a strong positive and significant correlation between total SOD activity, and H₂O₂ production in all tested NSAIDs (Fig. 6a-d). Mn-SOD activity demonstrated positive and significant correlations with H₂O₂ and total SOD activity for FFA and IBU, with Fe-SOD for NBT, IBU, and NPX, and also with *FSD1* for NPX (Fig. 6a-d). A similar relationship was found between *MSD5* and *FSD1* transcripts in the case of FFA, NBT, and NPX (Fig. 6a, b and d). Negative correlations were observed only for IBU between *MSD3* and H₂O₂, Fe-SOD, Mn-SOD, and total SOD activity, although only the correlation with Fe-SOD activity reached statistical significance.

### APX activity and gene expression

**Fig. 3:**
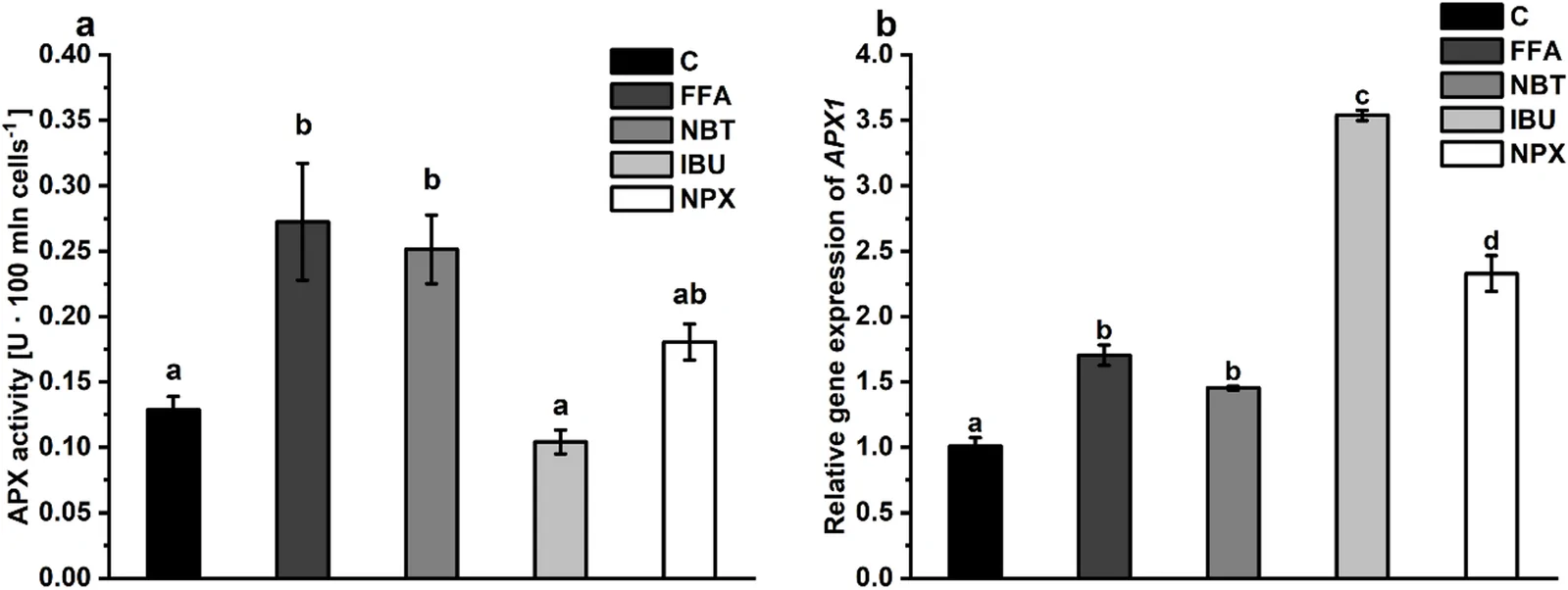
Effect of flufenamic acid (FFA), nabumetone (NBT), ibuprofen (IBU), and naproxen (NPX), applied at EC50 concentrations on the activity of ascorbate peroxidase (APX, **a)**, and relative gene transcription level of *APX1* **(b)** in *C. reinhardtii* cells. Data are presented as averages ± SE. Different letters indicate significant differences (*p* ≤ 0.05) between groups (ANOVA followed by the Tukey’s HSD test).

We also examined ascorbate peroxidase activity, a key enzyme involved in the decomposition of hydrogen peroxide produced in chloroplasts^26^. APX activity in untreated *C. reinhardtii* cells was about 0.13 U/ 100 million cells (Fig. 3a). The activity of APX was the lowest in IBU-treated cells, where it accounted for 81% of the control and was not statistically different (Figs. 3a and 5). All other pharmaceuticals tested increased APX activity in *C. reinhardtii* cells. The increase in APX activity was the least pronounced for NPX, reaching 140% of the control cells (Figs. 3a and 5). Meanwhile, the highest significant activity levels were recorded for FFA and NBT, achieving 212%, and 195% of the activity observed in untreated cells (Figs. 3a and 5). The expression of the gene encoding ascorbate peroxidase was also analyzed. All NSAIDs used in the study increased the relative transcript abundance of the *APX1* gene (Fig. 3b). The obtained values were 144% (NBT), 169% (FFA), 231% (NPX), and 351% (IBU) of the control (Figs. 3b and 5). Significant positive correlations were observed only for *APX1* with *MSD5* for IBU and with *MSD3* for NBT (Fig. 6b, c). Compared with *APX1*, *APX2* and *APX4* showed more variable expression profiles and did not allow clear interpretation of treatment-related changes. Therefore, further interpretation focused on *APX1*.

### CAT activity and gene expression

**Fig. 4:**
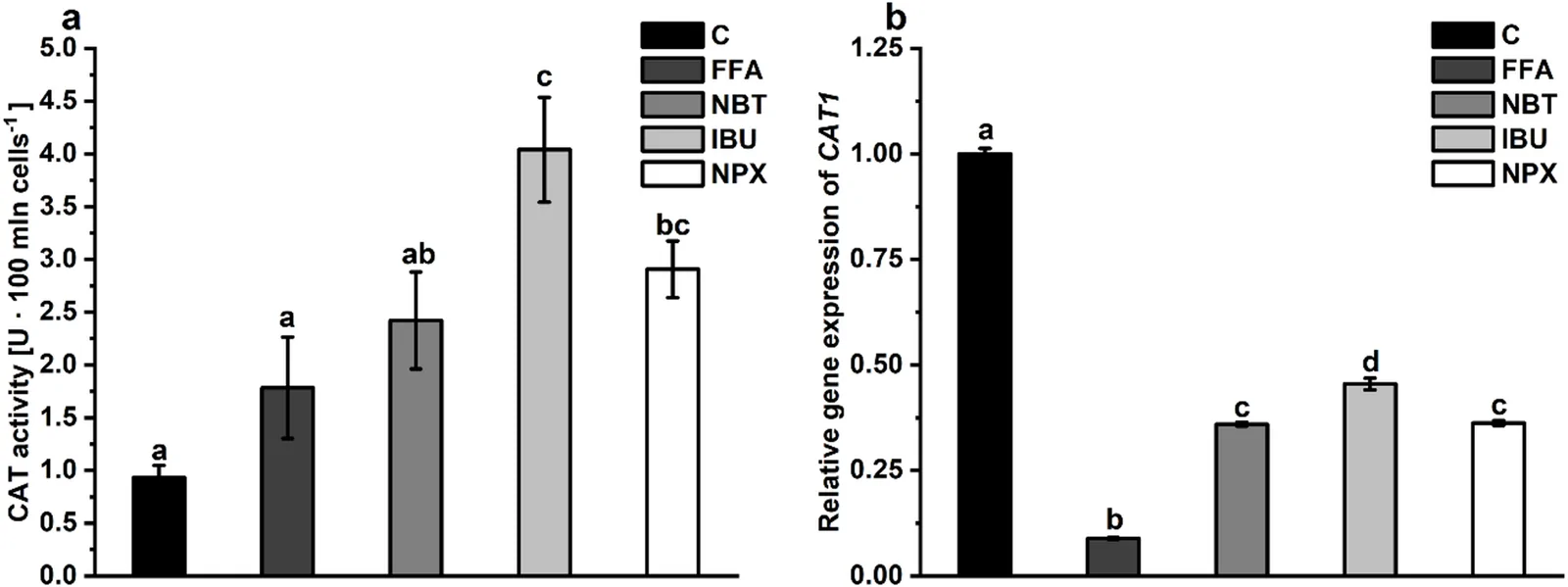
Effect of flufenamic acid (FFA), nabumetone (NBT), ibuprofen (IBU), and naproxen (NPX), applied at EC50 concentrations on activity of catalase (CAT, **a)**, and relative gene transcription level of *CAT1* **(b)** in *C. reinhardtii* cells. Data are presented as averages ± SE. Different letters indicate significant differences (*p* ≤ 0.05) between groups (ANOVA followed by the Tukey’s HSD test).

**Fig. 5:**
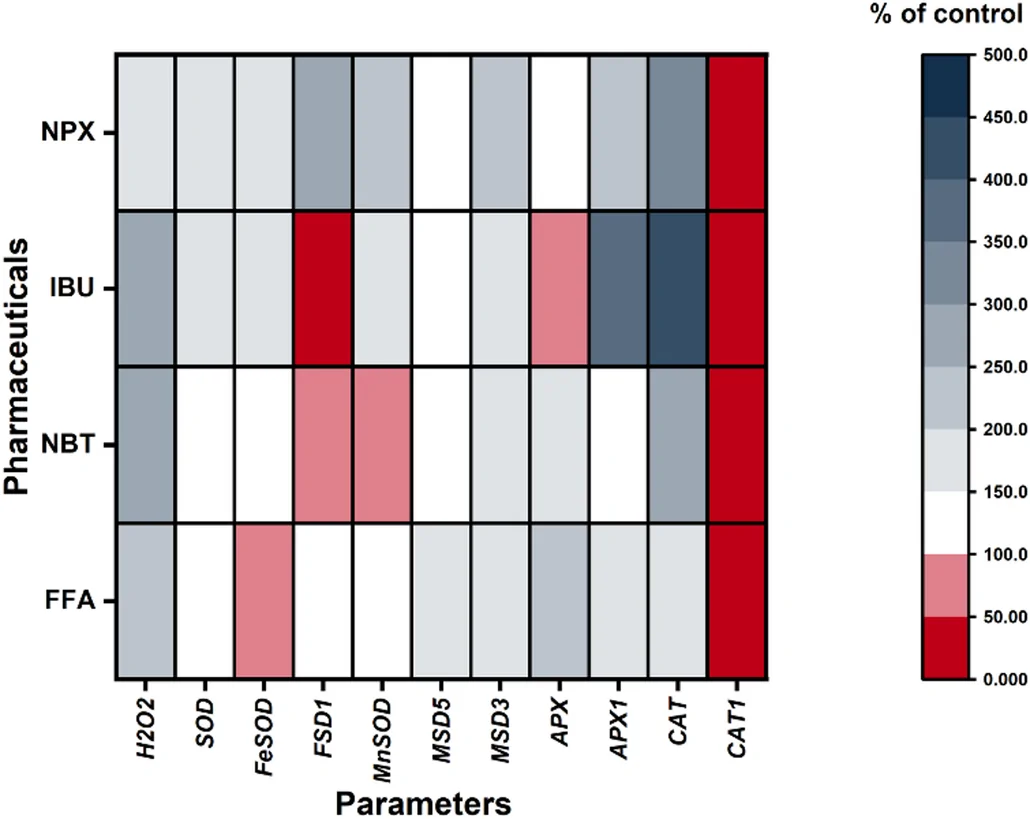
Overview of NSAIDs effect on analyzed parameters, relative to control values.

**Fig. 6:**
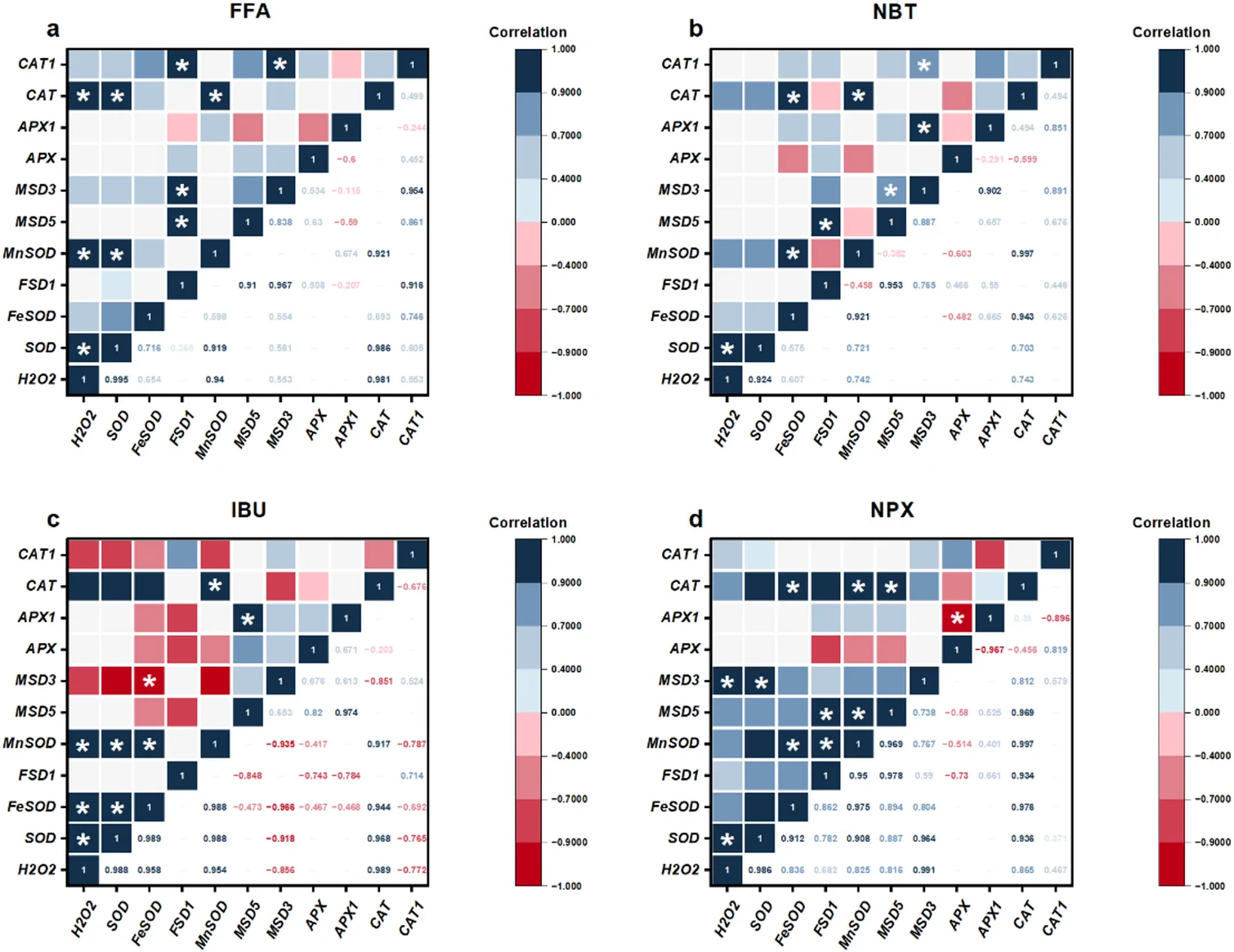
Pearson correlation matrices between H_2_O_2_ production, antioxidant enzyme activity, and gene expression in *C. reinhardtii* cells treated with FFA **(a)**, NBT **(b)**, IBU **(c)**, and NPX **(d)**. Positive correlations are shown in blue and negative correlations in red; color intensity is proportional to the correlation coefficient; white stars indicate statistically significant correlations (*p* ≤ 0.05). Grey squares indicate no linear relationship among the analyzed parameters.

The activity of catalase, which is involved in the decomposition of H_2_O_2_ into O_2_ and H_2_O outside chloroplasts, was increased in *C. reinhardtii* cells treated with each of the tested NSAIDs. In FFA- and NBT-treated cells, CAT activity reached 191%, and 259% of the control, respectively (Figs. 4a and 5). Treatment with IBU, and NPX resulted in a significant increase in the activity of this enzyme, reaching 432%, and 311% of the level observed in untreated cells, respectively (Figs. 4a and 5). Analysis of *CAT1* transcript abundance encoding catalase demonstrated the opposite effect to the activity of CAT observed in *C. reinhardtii* cells. All tested NSAIDs caused a significant decrease in the relative transcript levels of *CAT1* (Figs. 4b and 5). This value ranged from 9% to 46% of the level obtained in the control group (Figs. 4b and 5). Interestingly, all tested substances exhibited a significant and very strong positive correlation between CAT activity and Mn-SOD activity. Moderate, strong, and very strong correlations were also observed between CAT activity and H₂O₂ production, total SOD, and Fe-SOD activity in the cells exposed to the tested NSAIDs, although not all were statistically significant (Fig. 6a-d). We also detected a very strong positive correlation between *CAT1* and *FSD1* and *MSD3* levels in cells treated with FFA. A strong correlation between *CAT1* and *MSD3* transcript was also observed after NBT treatment (Fig. 6a, b).

**Fig. 7:**
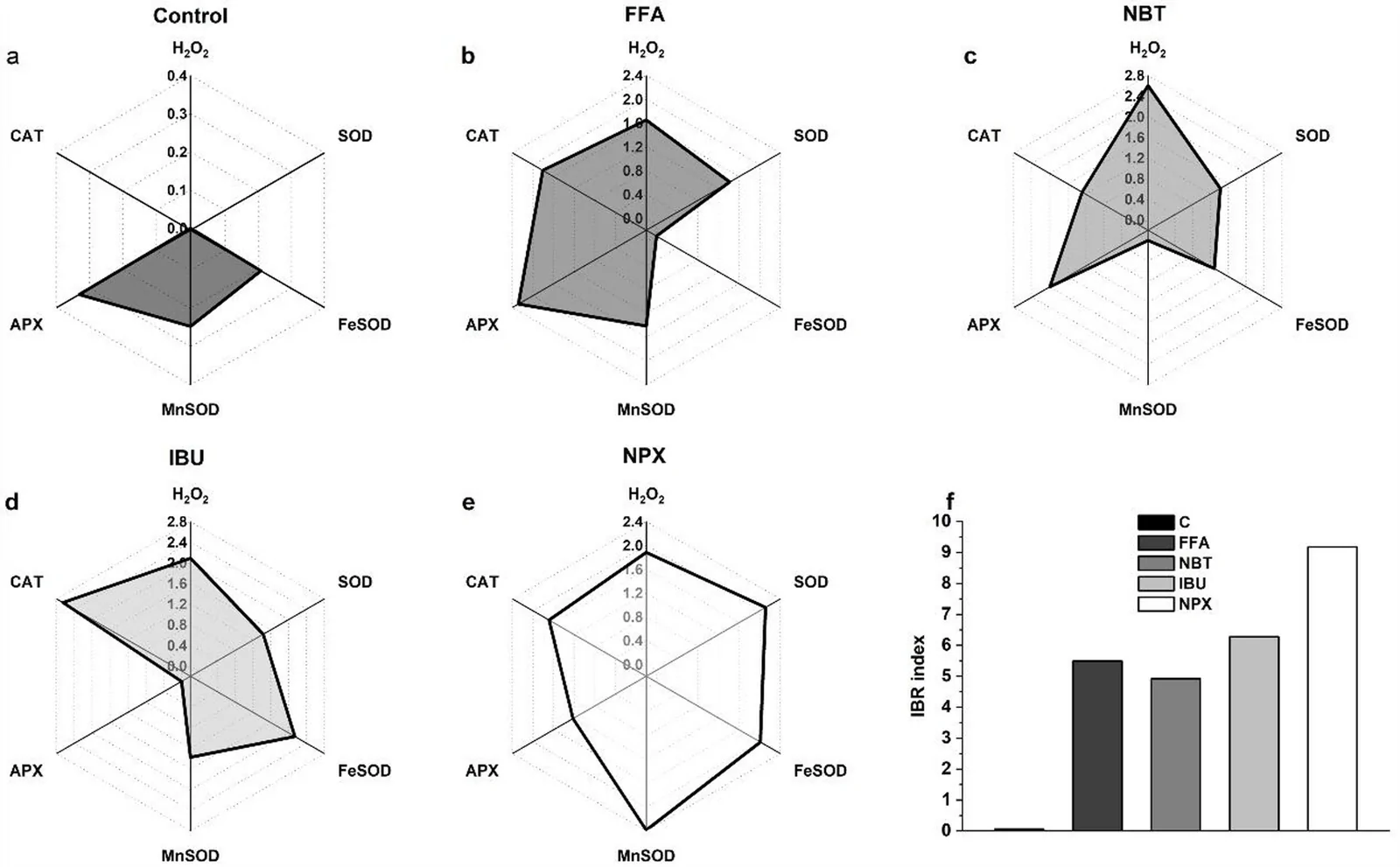
Biomarker star plots **(a-e)** and integrated biomarker response (IBR) index **(f)** of *C. reinhardtii* cells exposed to FFA, NBT, IBU, and NPX, applied at EC50 concentrations. H₂O₂- hydrogen peroxide production, SOD- total SOD activity, Fe-SOD- iron superoxide dismutase activity, Mn-SOD- manganese superoxide dismutase activity, APX- ascorbate peroxidase activity, CAT- catalase activity.

### Integrated Biomarkers Response (IBR)

The IBR analysis allowed for a comprehensive evaluation of the antioxidant system activity of *C. reinhardtii* cells treated with the tested NSAIDs (Fig. 7). We observed that with increasing NSAID toxicity (EC50), the IBR index value decreased (Fig. 7a). Among the tested biomarkers, APX and CAT showed the highest biological effects in cells treated with FFA (Fig. 7b). In the case of NBT, the production of H₂O₂ and APX activity played the most important role in IBR (Fig. 7c). The most sensitive biomarkers in cells treated with IBU were CAT and Fe-SOD (Fig. 7d). Interestingly, the highest S-values observed for the activity of SOD and its isoforms indicate that these biomarkers were most involved in the response of *C. reinhardtii* cells to NPX (Fig. 7e).

## Discussion

Abiotic stress caused by the exposure of living organisms to toxic substances is a major factor triggering the release of reactive oxygen species (ROS), potentially leading to oxidative stress when scavenging mechanisms are insufficient^27^. This research compared the antioxidant response of *C. reinhardtii* to selected NSAIDs, such as FFA, NBT, IBU, and NPX, and proved that each of the selected NSAIDs caused oxidative stress in *C. reinhardtii* cells, but the mechanisms involved in minimizing their negative effects differ.

Firstly, all tested NSAIDs increased H_2_O_2_ production in *C. reinhardtii* cells, confirming the induction of redox imbalance, growth inhibition, and reduced photosynthesis efficiency. This result is in line with our previous work focusing on the toxicity of diclofenac (DCF) to *C. reinhardtii*, where the amount of H_2_O_2_ produced by these microalgae was 200% of the control^12^. In this work, we observed that FFA, NBT, NPX, and IBU caused a similar or even greater overproduction of H_2_O_2_. This underscores the potential for these common NSAIDs to induce significant oxidative stress in aquatic photoautotrophs. Moreover, our observations align with the results of other research teams focusing on the effects of pharmaceuticals on ROS levels in algal cells, including increased superoxide anion production in *C. reinhardtii* cells treated with ibuprofen and the antibiotic oxytetracycline^14^. *Chlorella sp*. and *Desmodesmus spinosus* are other freshwater microalgae in which increased levels of ROS (hydrogen peroxide) have been observed after treating cells with amoxicillin, ciprofloxacin, tetracycline, paracetamol, ketoprofen, and diclofenac^28^. This strongly suggests that ROS overproduction is a common response of green algal cells to pharmaceutical-induced stress. Therefore, investigating cells´ capacity to scavenge ROS seemed to be a sensible choice for the research. The chemical properties of many NSAIDs, particularly their ability to perturb mitochondrial oxidative phosphorylation or interfere with photosynthetic electron flow^6,17,29^, suggest that multiple compartments (chloroplasts, mitochondria, peroxisomes) can serve as ROS sources during NSAIDs exposure. This compartment-specific ROS generation indicates that *C. reinhardtii* must activate a coordinated antioxidant response spanning several organelles to maintain redox homeostasis.

Secondly, the observed increases in H₂O₂ were accompanied by strong activation of the superoxide dismutases (SODs), which are often described as the first-line of defense in stressed cells. Total SOD activity increased significantly in IBU- and NPX-treated cells (Fig. 2a) and was strongly positively correlated with H₂O₂ production under all treatments. This confirms that SOD activity is tightly coupled to ROS accumulation; it was also shown using Pearson correlation matrices (Fig. 6), and is in line with the literature^30,31^.

The part of our study examining the expression of SOD-encoding genes, and the activity of SOD isoforms was particularly noteworthy, as studies analyzing SOD in NSAID-treated algae cells are scarce, and usually report only a total SOD activity. Such limited SOD-level analyses were reported, for example, in *Desmodesmus obliquus* exposed to ibuprofen, aspirin, and ketoprofen, where oxidative stress markers were measured, but isoenzyme-specific SOD responses were not assessed^6^. Similarly, studies on NSAIDs toxicity to *Chlorella* and *Desmodesmus* species have focused mainly on cellular stress biomarkers and total antioxidant enzyme activities, without resolving individual SOD isoforms^28^.

In this research, we found that the SOD response was highly isoform-specific at the isoenzyme level. Mn-SOD activity increased markedly after NPX exposure (~ 223% of control), indicating a strong involvement of mitochondria, and possibly peroxisomes in ROS detoxification. In contrast, Fe-SOD, localized mainly in chloroplasts, showed moderate, non-significant increases in NBT-, IBU-, and NPX-treated cells (Fig. 2a; Tab. S4; Tab. S5). These findings highlight that NSAIDs induce compartment-dependent ROS production/detoxification, with NPX in particular stimulating mitochondrial/peroxisomal antioxidant defenses. This organelle-specific ROS formation agrees with observations that several NSAIDs disrupt photosynthetic processes and stimulate ROS production, as shown for DCF in *C. reinhardtii* and ibuprofen/ketoprofen in *Scenedesmus obliquus*^6,17^.

At the transcriptional level, the expression of *FSD1*, encoding the chloroplast-located iron superoxide dismutase (_chl_Fe-SOD)^32^, increased significantly after FFA and NPX treatment. A comparable effect on *FSD1* expression was observed previously in *C. reinhardtii* treated with DCF, which enhanced expression of photosynthesis-, and redox-related genes, including *FSD1*, in response to oxidative stress^17^. In the present study, in NPX-treated cells, we found a positive correlation between *FSD1* expression and _chl_Fe-SOD activity (Figs. 2b and 6d). Moreover, _chl_Fe-SOD activity was very strongly positively correlated with the activity of Mn-SOD (predicted to localize to the secretory pathway; _sp_Mn-SOD)^32^, and strongly but not statistically significantly correlated with expression of its genes (*MSD3*, *MSD5*,* FSD1***)** (Fig. 6a-d). Importantly, *MSD3* expression increased significantly in all NSAID-treated cultures, making it a promising molecular biomarker of NSAID-induced oxidative stress in *C. reinhardtii*. Similar NSAID-induced oxidative stress signaling has been documented for DCF in algae and other aquatic organisms, where oxidative stress responses and DNA damage are consistently reported under chronic NSAID exposure^33–35^. Moreover, correlations between *FSD1* expression and Mn-SOD activity, as well as between *MSD5* expression and Mn-SOD activity, indicate coordinated regulation of chloroplast and extrachloroplast SOD isoenzymes under NPX stress. Overall, these results demonstrate that *C. reinhardtii* activates a compartmentalized, isoform-specific SOD response, essential for early adaptation to NSAID-induced oxidative stress.

Thirdly, although SOD is considered the first line of defense because it converts superoxide into hydrogen peroxide, the subsequent hydrogen peroxide detoxification in *C. reinhardtii* primarily depends on two enzymes, namely chloroplast-localized ascorbate peroxidase (APX), and catalase (CAT), which function in peroxisomes, the endoplasmic reticulum, and the cytoplasm^36,37^. Our results indicated that APX activity increased in all treatments except IBU, with the strongest induction observed for FFA and NBT (Fig. 3a). NPX induced a moderate increase (to ~ 140% of control), whereas in IBU-treated cells, APX activity was comparable to the control. Despite these differences, *APX1* transcript levels increased significantly under all treatments, especially in IBU and NPX-treated cells. This highlights that APX is consistently upregulated at the transcriptional level, suggesting that chloroplasts are an early and sensitive target of NSAID-induced redox perturbation. This interpretation is further supported by the chlorophyll *a* fluorescence data and pigment analyses, which revealed clear, compound- specific alterations in PSII performance, electron transport, and content of chlorophylls and carotenoids (Tab. S6)^13^. Taken together, these changes may reflect a partial acclimatory adjustment of photosynthetic apparatus, potentially contributing to stress mitigation. On the other hand, discrepancies between transcript level and enzyme activity indicate post-transcriptional regulation of APX activity. Such transcript-activity mismatches are well documented for APX. For instance, Lopez et al.,^38^ reported that salt stress in *Raphanus sativus* caused an increase in APX activity without a proportional rise in APX mRNA levels. Various reasons for such discrepancies have been suggested in the literature. First, post-translational modifications (PTMs) regulate APX activity *in vivo*^39,40^. In higher plants and some green algae, cysteine S-nitrosylation enhances, whereas tyrosine nitration diminishes APX activity. These changes occur independently of transcript levels and integrate redox signals from ROS, RNS, and thiol-disulfide interchange^39,40^. An additional recognized control mechanism involves isoform diversity and processing, including alternative splicing of genes^40^. Furthermore, APX activity depends on cofactors/substrates (such as ascorbate availability) and on compartment-specific oxidative environment^41^. Given the highest H_2_O_2_ levels in NSAID-exposed cells, the self-inactivation of APX through its product-catalyzed reaction provides a mechanistic explanation for the observed divergence^42^. The above-mentioned reports may explain the observed discrepancy between APX transcript levels and enzyme activity in NSAIDs-treated algal cells, especially pronounced in the case of IBU treatment (Fig. 3a-b).

While considering the role of APX in the cell, it should be noted that beyond ROS detoxification, this enzyme plays several important functions. It is distributed across various cellular compartments, such as chloroplasts, cytosol, mitochondria, peroxisomes, and even the apoplast in plant and algal cells^40,43,44^, and participates in redox signaling that coordinates stress responses and cell development. The second important enzymatic component of stress signaling and mitigation machinery is catalase. In contrast to APX, CAT activity increased in all NSAID-treated cells, with the highest values observed for IBU and NPX treatments. Surprisingly, *CAT1* transcript levels decreased in all treatments (to 9–46% of control). This striking divergence between transcriptional suppression and enzymatic activity remains difficult to interpret due to the limited availability of literature data, and thus further study is needed. Some reports have suggested that a decrease in the expression of certain nucleus-encoded genes, including CAT, may be caused by a retrograde signal originating from the chloroplast^45^. While the enzymatic activity of CAT could be regulated post-transcriptionally through enzyme stabilization, activation, or post-translational modifications^46–48^. In our opinion, strong, positive and significant correlations between Mn-SOD and CAT activity in all NSAIDs treatment (Fig. 6a-b) indicate a functional coupling between mitochondrial/peroxisomal superoxide detoxification (Mn-SOD) and downstream H₂O₂ removal (CAT). This supports a model in which NSAIDs enhance mitochondrial/peroxisomal ROS production, triggering compensatory activation of CAT despite transcriptional downregulation.

Analysis of our results directs our attention to structurally similar drugs, namely NPX and NBT. Although NBT and NPX share similar chemical structures (methoxynaphthalene derivatives)^49^, their biological effects differed considerably. Despite both substances causing H₂O₂ overproduction (Fig. 1b), the antioxidative system response was different. NPX strongly activated both Fe-SOD and Mn-SOD (gene expression and activity), and greatly increased CAT activity (Figs. 2a-b and 4a), indicating a multi-compartment antioxidant response. NBT, despite generating high H₂O₂ levels, did not significantly increase SOD activity and instead triggered a stronger APX response (Figs. 1a, 2a and 3a), highlighting a chloroplast-centered reaction. Data reporting direct comparisons of the phytotoxic effects of NBT and NPX are scarce, but those that are available indicate higher toxicity of NPX than NBT^50^. In our opinion, this observation may be explained by the higher reactivity of NPX and its greater photosensitizing potential due to the electron-donating effect of the additional methyl group present in NPX. ROS are expected to be formed at different steps during the photodegradation pathways of both drugs, which give rise to their effects on biomolecules, such as lipid peroxidation^51^.

Given the complex and not yet fully understood of NSAIDs` effects, we decided to analyze IBR, which provides a comprehensive evaluation of the integrated antioxidant system activity. The IBR analysis (Fig. 7) revealed that the IBR index decreased with increasing NSAID toxicity (EC50), indicating that highly toxic compounds reduce the overall efficiency of antioxidant adaptation in *C. reinhardtii*. Particularly, for FFA, APX, and CAT exhibited the highest contributions to the stress response, whereas for NBT, H₂O₂ production and APX activity had the largest biological effects. For IBU, CAT, and Fe-SOD were most responsive, and for NPX, SOD isoenzymes had the strongest influence (highest S-values). Thus, these results suggest that the most successful adaptive strategy, characteristic of less toxic compounds such as IBU and NPX, involves simultaneous activation of SOD isoenzymes and strong CAT activity, supporting efficient removal of both superoxide and hydrogen peroxide. Treatments with more toxic compounds (NBT, FFA) were mainly characterized by APX activation, which indicates a strong shift to chloroplast detoxification pathways, probably reflecting stronger chloroplast-targeted stress. To our knowledge, no published studies have reported NSAID-specific antioxidant-response patterns that could explain the differential activation of SOD/CAT versus APX pathways observed here. Thus, our findings provide a novel and unique insight into how chemically diverse NSAIDs shape compartment-specific redox strategies.

## Conclusions and perspectives

In conclusion, this study offers a comprehensive analysis of the antioxidant response in *C. reinhardtii* cells exposed to selected NSAIDs, which form a significant group of aquatic contaminants. Our findings also highlight the need for monitoring NSAID-induced oxidative stress in microalgae and their potential as indicators of pharmaceutical pollution. It is also proved that each of the selected NSAIDs caused oxidative stress in *C. reinhardtii* cells, but the mechanisms involved in minimizing their negative effects differ. Therefore, the key outcomes include: (i) identification of *MSD3* transcript level as a universal molecular biomarker of NSAID stress; (ii) evidence for compartment-specific adaptation, with APX serving as the primary chloroplast-localized H₂O₂ scavenger, particularly under FFA and NBT exposure, and with CAT playing a dominant role in extra chloroplast compartments, especially under IBU and NPX exposure; and (iii) recognition that NSAIDs induce compound-specific antioxidant signatures, reflecting their chemical properties. We also recommend that future studies should investigate subcellular redox dynamics using compartment-specific ROS reporters. Proteomic analysis is also recommended to reveal post-translational modifications of APX, CAT, and SOD proteins. Additionally, examining a wider range of pharmaceuticals and environmental mixtures to map chemotype-specific adaptation strategies would be highly valuable.

## Materials and methods

### Tested compounds

In this study four substances belonging to the group of non-steroidal anti-inflammatory drugs (NSAIDs) were examined, namely ibuprofen (IBU, 4-Isobutyl-α-methylphenylacetic acid, 99% purity, CAS number 15687-27-1), flufenamic acid (FFA, N-(3-[Trifluoromethyl]phenyl) anthranilic acid, 97% purity, CAS number 530-78-9), nabumetone (NBT, 4-(6-Methoxy-2-naphthyl)-2-butanone; 97% purity, CAS number 42924-53-8), and naproxen (NPX, (S)-6-Methoxy-α-methyl-2-naphthaleneacetic acid sodium salt, 98% purity, CAS number: 26159-32-2). All substances were purchased from Alfa Aesar (Ward Hill, Massachusetts, USA). Ibuprofen and naproxen were dissolved in ddH_2_O to obtain the stock solutions of 100 mg/mL. For preparing stock solutions of FFA (70 mg/mL) and NBT (100 mg/mL), DMSO (dimethyl sulfoxide, analytical grade, BioShop, Canada) was used as the carrier solvent. In the experiments, where FFA and NBT were used, the final DMSO concentration did not exceed 0.1% (v/v). This DMSO concentration has been verified in our laboratory, and previous studies have shown that DMSO at this level does not affect the vital parameters of the green alga *C. reinhardtii*^12,52–54^. NSAID solutions were added to the HSM medium to obtain the desired concentrations (see 5.2).

### Algae cultures and treatment

*C. reinhardtii* (CC-1690, WT) was purchased from the Chlamydomonas Resource Center (University of Minnesota, Minnesota, USA). The algae were transferred to a liquid HSM medium from slants used for long-term storage^55^ and pre-cultured for several days at 30 ± 1 ˚C, with continuous bubbling with sterilized air (bacteriological filter 0.2 mm PTFE; Sartorius, Germany), enriched with 2.5% CO_2_ (v/v), and at white fluorescent light (125 ± 5 µmol photons/m^2^ × s)^53^. The pre-cultures which were at the logarithmic growth phase were used to establish experimental sets for acute toxicity testing. The appropriate volume of cell suspension taken from pre-culture was mixed with HSM to achieve an initial algal population density of 0.5 × 10^6^ cells/mL, and divided into several culture vessels (200 mL volume each)^25,53^. The cultures were treated for 24 h with FFA, NBT, IBU, and NPX at 35.47, 5.75, 87.04, or 137.44 mg/L, respectively. The concentrations used represent nominal EC50 values that were determined from population growth inhibition curves, for FFA and NBT^13^, for IBU and NPX the equations are provided in the Supplement (see Text S1, Fig. S1, and Tab. S1). Cultures without the tested chemicals were used as controls. At the beginning of the experiment (0 h) and after 24 h of cultivation the algae cells were collected for analysis, as described below.

To unify the results to comparable units, the population density was determined in each experimental variant. The number of cells was counted using a particle counter Z2 (Beckman Coulter, USA), run by dedicated software. Additional measurements of chlorophyll *a* fluorescence *in vivo* and photosynthetic pigments content were analyzed, as described in the supplementary materials (Text S2).

### Hydrogen peroxide production by cells

The amount of hydrogen peroxide produced by microalgae cells was determined using the Amplex UltraRed^®^ fluorochrome (Molecular Probes, USA), according to the established protocol^56^. Briefly, the algae cells suspension was centrifuged at 291 RCF for 5 min., at room temperature, the cells’ pellet was discarded, and supernatant was used for H_2_O_2_ estimation. 50 µL of the supernatant was placed in a 96-well black plate, and mixed with a 50 µL reaction mixture comprising 10 mM Amplex UltraRed, and 10 U/mL horseradish peroxidase in 0.1 M potassium phosphate buffer (pH 7.0). For the preparation of fortified samples, a freshly prepared H_2_O_2_ solution (stabilized, analytical grade, Sigma, USA) of a concentration of 3 nmol/mL was used. Fortified samples were prepared by introducing hydrogen peroxide of known concentration into the supernatant obtained as described above, followed by the addition of the reaction mixture. The samples were incubated in the darkness for 25 min. at 25 ˚C, the fluorescence was measured at λ_ex_ = 560 nm and λ_em_ = 585 nm using a Varioskan Flash-Spectral Scanning Multimode Microplate Reader (ThermoFisher Scientific, USA). The concentration of H_2_O_2_ was expressed as the amount of compound produced by millions of cells and calculated using equation:

$${\rm{C }}{{\rm{H}}_{\rm{2}}}{{\rm{O}}_{\rm{2}}} = {\rm{ }}\left[ {{\rm{ST }} \times {\rm{ Fscorr}}} \right]/{\rm{ }}\left[ {\left( {{\rm{Fsscorr }}{-}{\rm{ Fscorr}}} \right){\rm{ }} \times {\rm{ Sv}}} \right]{\rm{ }}\left[ {{\rm{nmol}}/{\rm{uL}}} \right],$$

where:

ST – the amount of H_2_O_2_ spiked in the well (nmol); Fscorr – corrected sample fluorescence reading (unspiked well); Fsscorr – corrected spiked sample fluorescence reading (spiked well); Sv – sample volume (µL) added into the well.

### The activity of antioxidative enzymes

All chemicals used for the enzymes’ activity analyses were of analytical grade purity (Sigma-Aldrich, Missouri, USA). To analyze the activity of antioxidative enzymes, 100 mL of algae suspension was collected by centrifugation (5 min., 3 000 × g), and the sedimented material was re-suspended in 1 mL of appropriate extraction buffer. For superoxide dismutase (SOD) and catalase (CAT), 0.05 M potassium phosphate buffer (pH 7.0) was used, while for ascorbic peroxidase (APX), 0.05 M phosphate buffer (pH 7.6), containing 0.86 mM AsA (ascorbic acid), 2.4 mM EDTA (ethylenediaminetetraacetic acid), 20% sorbitol and 2% PVP (polyvinylpyrrolidone) was used. Briefly, the pellet was homogenized with glass beads (30 s, 4.5 RPS) in 1 mL of the appropriate buffer using FastPrep^®^-24 (MP Biomedicals, USA), after which the samples were centrifuged (20 min., 20.000 × g, at 4 ˚C). The obtained supernatants were used to determine protein concentration and enzyme activities.

Protein concentration was examined according to the method of Bradford^57^. Briefly, 300 µL of Bradford reagent was added to 10 µL of sample or bovine albumin standard and incubated for 10 min. in room temperature. Measurements were made at a wavelength of 595 nm using a Varioskan Flash-Spectral Scanning Multimode Microplate Reader (ThermoFisher Scientific, USA). The protein concentration in the analyzed supernatant samples was calculated using the equation derived from the standard curve. The results were used for enzyme activity calculations.

The method involving the reduction of nitroblue tetrazolium (NBT) was used to determine the activity of SOD isoforms^58,59^. Briefly, non-denaturing polyacrylamide gel electrophoresis (PAGE) was performed, and the resulting gels were immersed in a 2.45 mM NBT solution for 20 min., followed by immersion in a solution containing 28 mM TEMED and 2.8 × 10^− 6^ M riboflavin in 36 mM potassium phosphate buffer at pH 7.8 for 15 min. The gels were exposed to illumination until a uniform blue color was obtained, with areas containing SOD remaining colorless. SOD isoforms were resolved on the gels^60^. The activity of individual SOD isoform bands was determined by comparing the relative band intensity in mm² to that of the Cu/Zn-SOD reference pattern with a defined activity of 4.4 U/mg protein (Sigma-Aldrich, Missouri, USA). Densitometric analysis was performed using ImageLab 6.0 (Bio-Rad Laboratories, Inc., California, USA).

APX activity was determined according to the method of Nakano and Asada^61^, as described in Aksmann et al.,^54^ with a slight modification. A reaction mixture containing 50 mM phosphate buffer (pH 7.0), 18 mM pyrogallol, and 0.1 mM H_2_O_2_ was prepared. To 50 µL of enzyme extract, 150 µL of the reaction mixture was added, and the decrease in absorbance at 430 nm corresponding to pyrogallol oxidation was monitored for 1 min at 25 ˚C (by Hitachi U-2900 spectrophotometer, Japan). Enzyme activity was quantified using the molar extinction coefficient for pyrogallol (ε = 2.47 mM^− 1^ cm^− 1^), and the results were expressed as U per 100 million cells.

CAT activity was determined spectrophotometrically (Hitachi U-2900, Japan), as described in Aksmann et al.,^54^. Briefly, after adding the reaction mixture (50 mM sodium phosphate buffer pH = 7.0, 10 mM H_2_O_2_) to the enzyme extract, the decrease in absorbance (λ = 240 nm) was monitored^62^. Results were expressed as U per 100 million cells.

### RNA extraction and RT-qPCR

Transcript levels of selected genes related to oxidative stress in NSAIDs-treated cells were determined, as described by Aksmann et al., and Pokora et al.,^54,63,64^. Total RNA was isolated using the Total RNA Mini Kit (A&A Biotechnology, Poland) according to the manufacturer’s instructions, after centrifugation of algal cells (5 min, 3000 × g). Reverse transcription (RT) was carried out using an iScript™ cDNA Synthesis Kit 170–8891 (Bio-Rad Laboratories, Inc.) from 200 ng of total RNA with oligohexamer (dT) primers. Real-time PCR was performed using DNA-binding dye SYBR Green I (iTaq™ Universal SYBR^®^ Green Supermix, Bio-Rad Laboratories, Inc.), following the manufacturer’s protocol. Gene-specific primer pairs of *FSD1* (iron superoxide dismutase Fe-SOD), *MSD3*, *MSD4*, *MSD5* (isoforms of manganese superoxide dismutase Mn-SOD), *CAT1* (catalase, CAT), and *APX1*,* APX2*,* APX4* (isoforms of ascorbate peroxidase, APX) are listed in Table S3. The *18 S* transcript was used to standardize the results as an internal quantifying control. The levels of relative gene expression were calculated using Pfaffl method^65^. All PCR reactions and data analysis were performed on the CFX96 Touch™ Real-Time PCR Detection System using CFX Maestro™ Software (Bio-Rad Laboratories, Inc., California, USA).

### In silico prediction of enzymes’ subcellular localization

Computational predictions of the subcellular localization of *CAT1*, *APX1*, APX2, APX4, *FSD1*, *MSD3*, *MSD4*, and *MSD5* gene products were performed using web-based interfaces provided by each prediction program. The sequences of the nucleotide, or protein from algae, were retrieved in the FASTA format from the NCBI (https://www.ncbi.nlm.nih.gov/). Subcellular localizations were predicted using online tools such as UniPort^66^, deeploc2.1^67^, and PredAlgo^68^.

### Statistical analysis

The presented data were prepared in at least three independent experiments, each including a minimum of three biological replicates per treatment and two technical replicates. Microsoft Excel (Microsoft, 2023, USA), Origin Pro 9.6 (OriginLab Corp., USA), and Statistica 9.0 (StatSoft Europe, Germany) were used for experimental data processing, statistical analysis and graphics preparation. The data were expressed as the means ± SE. Data were analyzed by one-way ANOVA or Kruskal-Wallis test, followed by the Tukey or Dunn’s post-tests. Statistical analyses were performed at a probability value of < p 0.05, where the differences are marked using letters. The same software was used to calculate the Pearson correlation matrix to determine the relationship between H₂O₂ production, the activity of antioxidant enzymes, and the expression of the analyzed genes. The integrated biological response index (IBR) was also calculated for the control group and algae treated with NSAIDs, following the method^69^. The H_2_O_2_ production, and activity of SODs isoforms, CAT, and APX were selected as the biomarkers to analyze the antioxidant response of *C. reinhardtii* cells.

## Supplementary Information

Below is the link to the electronic supplementary material.


Supplementary Material 1


## Data Availability

All data generated or analysed during this study are included in this published article and its Supplementary Information files. Raw datasets are available from the corresponding author upon reasonable request.

## References

[1] Daughton, C. G. Cradle-to-cradle stewardship of drugs for minimizing their environmental disposition while promoting human health. I. Rationale for and avenues toward a green pharmacy. *Environ. Health Perspect.* **111**, 757 (2003). 12727606 10.1289/ehp.5947 PMC1241487

[2] Hejna, M., Kapuścińska, D. & Aksmann, A. Pharmaceuticals in the aquatic environment: a review on eco-toxicology and the remediation potential of algae. *Int. J. Environ. Res. Public. Health*. **19**, 7717 (2022). 35805373 10.3390/ijerph19137717 PMC9266021

[3] Świacka, K., Michnowska, A., Maculewicz, J., Caban, M. & Smolarz, K. Toxic effects of NSAIDs in non-target species: A review from the perspective of the aquatic environment. *Environ. Pollut*. **273**, 115891 (2021). 33497943 10.1016/j.envpol.2020.115891

[4] Parolini, M. Toxicity of the non-steroidal anti-inflammatory drugs (NSAIDs) acetylsalicylic acid, paracetamol, diclofenac, ibuprofen and naproxen towards freshwater invertebrates: A review. *Sci. Total Environ.* **740**, 140043 (2020). 32559537 10.1016/j.scitotenv.2020.140043

[5] Madikizela, L. M., Botha, T. L., Kamika, I. & Msagati, T. A. M. Uptake, occurrence, and effects of nonsteroidal anti-inflammatory drugs and analgesics in plants and edible crops. *J. Agric. Food Chem.* **70**, 34–45 (2022). 34967604 10.1021/acs.jafc.1c06499

[6] Wang, H. et al. Photosynthetic toxicity of non-steroidal anti-inflammatory drugs (NSAIDs) on green algae *Scenedesmus obliquus*. *Sci. Total Environ.* **707**, 136176 (2020). 31972914 10.1016/j.scitotenv.2019.136176

[7] Scranton, M. A., Ostrand, J. T., Fields, F. J. & Mayfield, S. P. *Chlamydomonas* as a model for biofuels and bio-products production. *Plant. J.* **82**, 523–531 (2015). 25641390 10.1111/tpj.12780 PMC5531182

[8] Singh, A. et al. An exploration of lipid remodeling by microalga *Chlamydomonas reinhardtii* in autotrophic and mixotrophic cultivation for bioenergy prospects. *Biomass Bioenerg*. **200**, 108015 (2025).

[9] Singh, A., Singh, S., Singh, S., Prasad, N. & Asthana, R. K. Harnessing bioenergy potential of *Chlamydomonas reinhardtii*: a comprehensive characterization and valorization of biomass towards energy conversion under circular bioeconomy. *Renew. Energy*. **240**, 122256 (2025).

[10] Ahn, J. W. & Choi, J. il. Recent advances in *Chlamydomonas reinhardtii* as a green factory for sustainable biofuels and bioproduct production. *N Biotechnol.* **93**, 362–377 (2026). 42070768 10.1016/j.nbt.2026.04.005

[11] Cleuvers, M. Mixture toxicity of the anti-inflammatory drugs diclofenac, ibuprofen, naproxen, and acetylsalicylic acid. *Ecotoxicol. Environ. Saf.* **59**, 309–315 (2004). 15388270 10.1016/S0147-6513(03)00141-6

[12] Majewska, M., Harshkova, D., Guściora, M. & Aksmann, A. Phytotoxic activity of diclofenac: Evaluation using a model green alga *Chlamydomonas reinhardtii* with atrazine as a reference substance. *Chemosphere* **209**, 989–997 (2018). 30114750 10.1016/j.chemosphere.2018.06.156

[13] Kapuścińska, D., Narajczyk, M., Liakh, I., Wielgomas, B. & Aksmann, A. Nabumetone and flufenamic acid pose a serious risk to aquatic plants: a study with *Chlamydomonas reinhardtii* as a model organism. *Chemosphere* **349**, 140853 (2024). 38052310 10.1016/j.chemosphere.2023.140853

[14] Seoane, M., Conde-Pérez, K., Esperanza, M., Cid, Á. & Rioboo, C. Unravelling joint cytotoxicity of ibuprofen and oxytetracycline on *Chlamydomonas reinhardtii* using a programmed cell death-related biomarkers panel. *Aquat. Toxicol.* **257**, 106455 (2023). 36841069 10.1016/j.aquatox.2023.106455

[15] Ding, T. et al. Biodegradation of naproxen by freshwater algae *Cymbella* sp. and *Scenedesmus quadricauda* and the comparative toxicity. *Bioresour Technol.* **238**, 164–173 (2017). 28433904 10.1016/j.biortech.2017.04.018

[16] Moreno-Sánchez, R. et al. Inhibition and uncoupling of oxidative phosphorylation by nonsteroidal anti-inflammatory drugs. *Biochem. Pharmacol.* **57**, 743–752 (1999). 10075080 10.1016/s0006-2952(98)00330-x

[17] Majewska, M. et al. Does diclofenac act like a photosynthetic herbicide on green algae? *Chlamydomonas reinhardtii* synchronous culture-based study with atrazine as reference. *Ecotoxicol. Environ. Saf.* **208**, 111630 (2021). 33396150 10.1016/j.ecoenv.2020.111630

[18] Zhong, X., Zhu, Y., Wang, Y., Zhao, Q. & Huang, H. Effects of three antibiotics on growth and antioxidant response of *Chlorella pyrenoidosa* and *Anabaena cylindrica*. *Ecotoxicol. Environ. Saf.* **211**, 111954 (2021). 33476846 10.1016/j.ecoenv.2021.111954

[19] Foyer, C. H. & Noctor, G. Oxidant and antioxidant signalling in plants: a re-evaluation of the concept of oxidative stress in a physiological context. *Plant. Cell. Environ.* **28**, 1056–1071 (2005).

[20] Wakao, S. & Niyogi, K. K. *Chlamydomonas* as a model for reactive oxygen species signaling and thiol redox regulation in the green lineage. *Plant. Physiol.* **187**, 687–698 (2021). 35237823 10.1093/plphys/kiab355 PMC8491031

[21] Pokora, W., Tułodziecki, S., Dettlaff-Pokora, A. & Aksmann, A. Cross talk between hydrogen peroxide and nitric oxide in the unicellular green algae cell cycle: how does it work? *Cells* 11, (2022). 10.3390/cells11152425 PMC9368121 35954269

[22] Xin, X., Huang, G. & Zhang, B. Review of aquatic toxicity of pharmaceuticals and personal care products to algae. *J. Hazard. Mater.* **410**, 124619 (2021). 33248823 10.1016/j.jhazmat.2020.124619

[23] Allen, M. D. et al. Manganese deficiency in *Chlamydomonas* results in loss of photosystem II and MnSOD function, sensitivity to peroxides, and secondary phosphorus and iron deficiency. *Plant. Physiol.* **143**, 263–277 (2007). 17085511 10.1104/pp.106.088609 PMC1761973

[24] Rajput, V. D. et al. Recent Developments in enzymatic antioxidant defence mechanism in plants with special reference to abiotic stress. *Biology* **10**, 10 (2021). 10.3390/biology10040267 PMC8066271 33810535

[25] Aksmann, A. et al. Time-dependent changes in antioxidative enzyme expression and photosynthetic activity of *Chlamydomonas reinhardtii* cells under acute exposure to cadmium and anthracene. *Ecotoxicol. Environ. Saf.* **110**, 31–40 (2014). 25193882 10.1016/j.ecoenv.2014.08.005

[26] Kuo, E. Y. H., Cai, M. S. & Lee, T. M. Ascorbate peroxidase 4 plays a role in the tolerance of *Chlamydomonas reinhardtii* to photo-oxidative stress. *Sci. Rep.* **10**, 1–12 (2020). 32764698 10.1038/s41598-020-70247-z PMC7414030

[27] Sachdev, S., Ansari, S. A., Ansari, M. I., Fujita, M. & Hasanuzzaman, M. Abiotic stress and reactive oxygen species: generation, signaling, and defense mechanisms. *Antioxidants* **10**, 277 (2021). 33670123 10.3390/antiox10020277 PMC7916865

[28] Gomaa, M., Zien-Elabdeen, A., Hifney, A. F. & Adam, M. S. Phycotoxicity of antibiotics and non-steroidal anti-inflammatory drugs to green algae *Chlorella* sp. and *Desmodesmus spinosus*: assessment of combined toxicity by Box–Behnken experimental design. *Environ. Technol. Innov.* **23**, 101586 (2021).

[29] Harshkova, D., Zielinska, E., Narajczyk, M., Kapusta, M. & Aksmann, A. Mitochondria dysfunction is one of the causes of diclofenac toxicity in the green alga *Chlamydomonas reinhardtii*. *PeerJ* 12, (2024). 10.7717/peerj.18005 PMC11365475 39221263

[30] Berwal, M. K., Ram, C. & Superoxide Dismutase A stable biochemical marker for abiotic stress tolerance in higher plants. *Abiotic and Biotic Stress in Plants* (2018).

[31] Rao, M. J. et al. Antioxidant Defense System in Plants: Reactive Oxygen Species production, signaling, and scavenging during abiotic stress-induced oxidative damage. *Horticulturae* **11**, 477 (2025).

[32] Blaby, I. K. et al. Genome-wide analysis on *Chlamydomonas reinhardtii* reveals the impact of hydrogen peroxide on protein stress responses and overlap with other stress transcriptomes. *Plant. J.* **84**, 974–988 (2015). 26473430 10.1111/tpj.13053 PMC4715741

[33] Pandey, P. K., Ajima, M. N. O., Kumar, K., Poojary, N. & Kumar, S. Evaluation of DNA damage and physiological responses in *Nile tilapia*, *Oreochromis niloticus* (Linnaeus, 1758) exposed to sub-lethal diclofenac (DCF). *Aquat. Toxicol.* **186**, 205–214 (2017). 28324828 10.1016/j.aquatox.2017.03.007

[34] Mirzaee, S. A. et al. The possible oxidative stress and DNA damage induced in diclofenac-exposed non-target organisms in the aquatic environment: a systematic review. *Ecol. Indic.* **131**, 108172 (2021).

[35] Touzout, N. et al. Understanding the ecotoxicity of diclofenac on the freshwater cyanobacterium arthrospira platensis through growth kinetics, primary biocomponents, oxidative metabolism, and molecular docking. *Water Air Soil. Pollut*. **237**, 119 (2025).

[36] Kato, N., Nelson, G. & Lauersen, K. J. Subcellular localizations of catalase and exogenously added fatty acid in *Chlamydomonas reinhardtii*. *Cells* **10**, (2021) 10.3390/cells10081940 PMC8391285 34440712

[37] Maruta, T., Sawa, Y., Shigeoka, S. & Ishikawa, T. Diversity and evolution of ascorbate peroxidase functions in chloroplasts: more than just a classical antioxidant enzyme? *Plant. Cell. Physiol.* **57**, 1377–1386 (2016). 26738546 10.1093/pcp/pcv203

[38] Lopez, F., Vansuyt, G., Casse-Delbart, F. & Fourcroy, P. Ascorbate peroxidase activity, not the mRNA level, is enhanced in salt-stressed *Raphanus sativus* plants. *Physiol. Plant.* **97**, 13–20 (1996).

[39] Caccamo, A. The role of ascorbate peroxidase 2 and protein cysteine modifications in the green alga *Chlamydomonas reinhardtii.* (2024). https://researchportal.vub.be/en/publications/the-role-of-ascorbate-peroxidase-2-and-protein-cysteine-modificat/

[40] Yoshimura, K. & Ishikawa, T. Physiological function and regulation of ascorbate peroxidase isoforms. *J. Exp. Bot.* **75**, 2700–2715 (2024). 38367016 10.1093/jxb/erae061

[41] Li, S. Novel insight into functions of ascorbate peroxidase in higher plants: more than a simple antioxidant enzyme. *Redox Biol.* **64**, 102789 (2023). 37352686 10.1016/j.redox.2023.102789 PMC10333675

[42] Hiner, A. N. P. et al. Kinetic study of the inactivation of ascorbate peroxidase by hydrogen peroxide. *Biochem. J.* **348**, 321 (2000). 10816425 PMC1221069

[43] Kaur, S. et al. Regulation of dual activity of ascorbate peroxidase 1 from *Arabidopsis thaliana* by conformational changes and posttranslational modifications. *Front. Plant. Sci.* **12**, 1027730 (2021). 10.3389/fpls.2021.678111 PMC8236860 34194454

[44] Madhusudhan, R., Ishikawa, T., Sawa, Y., Shigeoka, S. & Shibata, H. Post-transcriptional regulation of ascorbate peroxidase during light adaptation of *Euglena gracilis*. *Plant. Sci.* **165**, 233–238 (2003).

[45] Mukai, K. et al. Effects of light and hydrogen peroxide on gene expression of newly identified antioxidant enzymes in the harmful algal bloom species *Chattonella marina*. *Eur. J. Phycol.* **54**, 393–403 (2019).

[46] Zhang, Y., Wang, L. F., Li, T. T. & Liu, W. C. Mutual promotion of *LAP2* and *CAT2* synergistically regulates plant salt and osmotic stress tolerance. *Front. Plant. Sci.* **12**, 672672 (2021). 34177987 10.3389/fpls.2021.672672 PMC8220078

[47] Jiménez, A., Martí, M. C. & Sevilla, F. Oxidative post-translational modifications of plant antioxidant systems under environmental stress. *Physiol. Plant.* **177**, 70118 (2025). 10.1111/ppl.70118 PMC11837463 39968905

[48] Martí-Guillén, J. M., Pardo-Hernández, M., Martínez-Lorente, S. E., Almagro, L. & Rivero, R. M. Redox post-translational modifications and their interplay in plant abiotic stress tolerance. *Front. Plant. Sci.* **13**, 1027730 (2022). 36388514 10.3389/fpls.2022.1027730 PMC9644032

[49] NCBI. (2025). https://pubchem.ncbi.nlm.nih.gov/compound/4409

[50] Situmeang, D. & Suwitono, M. R. Predicting pollutant toxicity of over-the-counter (OTC) pain killers (Analgesic) pharmaceutical drug. in *11th International Scholars Conference*Universitas Advent Indonesia, Indonesia, (2024).

[51] Musa, K. A. K. & Eriksson, L. A. Theoretical study of the phototoxicity of naproxen and the active form of nabumetone. *J. Phys. Chem. A*. **112**, 10921–10930 (2008). 18834087 10.1021/jp805614y

[52] Aksmann, A. & Tukaj, Z. The effect of anthracene and phenanthrene on the growth, photosynthesis, and SOD activity of the green alga *Scenedesmus armatus* depends on the PAR irradiance and CO_2_ level. *Arch. Environ. Contam. Toxicol.* **47**, 177–184 (2004). 15386142 10.1007/s00244-004-2297-9

[53] Aksmann, A. & Tukaj, Z. Intact anthracene inhibits photosynthesis in algal cells: a fluorescence induction study on *Chlamydomonas reinhardtii cw92* strain. *Chemosphere* **74**, 26–32 (2008). 18980775 10.1016/j.chemosphere.2008.09.064

[54] Aksmann, A., Pokora, W., Baścik-Remisiewicz, A., Dettlaff-Pokora, A. & Tukaj, Z. High hydrogen peroxide production and antioxidative enzymes expression in the *Chlamydomonas reinhardtii cia3* mutant with an increased tolerance to cadmium and anthracene. *Phycological Res.* **64**, 300–311 (2016).

[55] Harris, E. H. *The Chlamydomonas sourcebook: introduction to Chlamydomonas and its laboratory use: Volume 1* (Academic, 2009).

[56] Hejna, M., Kapuścińska, D. & Aksmann, A. A sensitive and reliable method for the quantitative determination of hydrogen peroxide produced by microalgae cells. *J. Phycol.* **60**, 1356–1370 (2024). 39585191 10.1111/jpy.13524

[57] Bradford, M. M. A rapid and sensitive method for the quantitation of microgram quantities of protein utilizing the principle of protein-dye binding. *Anal. Biochem.* **72**, 248–254 (1976). 942051 10.1016/0003-2697(76)90527-3

[58] Davis, B. J. Disc electrophoresis – II method and application to human serum proteins. *Ann. N Y Acad. Sci.* **121**, 404–427 (1964). 14240539 10.1111/j.1749-6632.1964.tb14213.x

[59] Tukaj, Z. & Pokora, W. Individual and combined effect of anthracene, cadmium, and chloridazone on growth and activity of SOD izoformes in three *Scenedesmus species*. *Ecotoxicol. Environ. Saf.* **65**, 323–331 (2006). 16464497 10.1016/j.ecoenv.2005.12.001

[60] Beauchamp, C. & Fridovich, I. Superoxide dismutase: improved assays and an assay applicable to acrylamide gels. *Anal. Biochem.* **44**, 276–287 (1971). 4943714 10.1016/0003-2697(71)90370-8

[61] Nakano, Y. & Asada, K. Hydrogen peroxide is scavenged by ascorbate-specific peroxidase in spinach chloroplasts. *Plant. Cell. Physiol.* **22**, 867–880 (1981).

[62] Aebi, H. & Catalase *in vitro*. in *Methods in Enzymology* 105. 121–126Academic Press, (1984). 10.1016/s0076-6879(84)05016-3 6727660

[63] Pokora, W. et al. Changes in nitric oxide/hydrogen peroxide content and cell cycle progression: Study with synchronized cultures of green alga *Chlamydomonas reinhardtii*. *J. Plant. Physiol.* **208**, 84–93 (2017). 27894022 10.1016/j.jplph.2016.10.008

[64] Pokora, W., Aksmann, A., Baścik-Remisiewicz, A., Dettlaff-Pokora, A. & Tukaj, Z. Exogenously applied hydrogen peroxide modifies the course of the *Chlamydomonas reinhardtii* cell cycle. *J. Plant. Physiol.* **230**, 61–72 (2018). 30170242 10.1016/j.jplph.2018.07.015

[65] Pfaffl, M. W. A new mathematical model for relative quantification in real-time RT–PCR. *Nucleic Acids Res.* **29**, e45 (2001). 11328886 10.1093/nar/29.9.e45 PMC55695

[66] Bateman, A. et al. UniProt: the universal protein knowledgebase in 2023. *Nucleic Acids Res.* **51**, D523–D531 (2023). 36408920 10.1093/nar/gkac1052 PMC9825514

[67] Ødum, M. T. et al. DeepLoc 2.1: multi-label membrane protein type prediction using protein language models. *Nucleic Acids Res.* **52**, W215–W220 (2024). 38587188 10.1093/nar/gkae237 PMC11223819

[68] Tardif, M. et al. PredAlgo: a new subcellular localization prediction tool dedicated to green algae. *Mol. Biol. Evol.* **29**, 3625–3639 (2012). 22826458 10.1093/molbev/mss178

[69] Beliaeff, B. & Burgeot, T. Integrated biomarker response: a useful tool for ecological risk assessment. *Environ. Toxicol. Chem.* **21**, 1316–1322 (2002). 12069320

