# Supplementary material for: Differential antioxidant pathways displayed by Chlamydomonas reinhardtii exposed to selected NSAIDs
Source: Sci Rep. 2026 Jun 15;16:27289. doi: 10.1038/s41598-026-57688-8 (PMC13530185; doi:10.1038/s41598-026-57688-8)
Supplement: Supplementary file 1 [file 41598_2026_57688_MOESM1_ESM.docx]

Supplementary Material for:

Differential antioxidant pathways displayed by *Chlamydomonas reinhardtii* exposed to selected NSAIDs

Dominika Kapuścińska^1^, Monika Hejna^2*^, Wojciech Pokora^1^, Anna Aksmann^1*^

*^1^**University of Gdańsk, Faculty of Biology, Department of Plant Experimental Biology and Biotechnology, Wita Stwosza 59, 80-308 Gdańsk, Poland.*

*^2^ University of Warsaw, Faculty of Biology, Institute of Microbiology, Department of Bacterial Physiology, Ilji Miecznikowa 1, 02-096 Warsaw, Poland.*

* Corresponding author: m.hejna@uw.edu.pl, anna.aksmann@ug.edu.pl

**The supporting data contains:**

**Text S1. Determination of effective concentrations for ibuprofen and naproxen.**

Algae were exposed to nominal ibuprofen (IBU) concentrations of 17.5, 30.6, 53.6, 93.8, 164.1 mg * dm^-3^ and naproxen (NPX) concentrations of 30, 52.5, 91.9, 160.8, 281.4 mg * dm^-3^ applied at the beginning of the experiment (t0). Cultures without the tested chemicals served as controls. After 24 h (t24) cultures were sampled for population density measurements. The effective concentrations for IBU and NPX were calculated by regression analysis based on linear models of the dependency between the relative population density (cells number) versus the pharmaceutical concentrations using the following equation: y = a + b*x. where a is intercept and b is slope. The values of these variables are presented in the table S1.

**Table S1.** Variables in the equations for determining EC values for IBU and NBT.

| **Variant** | **a** | **b** |
| --- | --- | --- |
| **IBU** | 89.23517 ± 1.24703 | -0.45079 ± 0.01305 |
| **NPX** | 79.42202 ± 2.35328 | -0.21407 ± 0.01436 |

**Figure S1.** Concenration-dependent growth inhibition curves of *C. reinhardtii* exposed to ibuprofen and naproxen for 24 h. Data are presented as averages (± SE).

**Table S2.** Specific growth rate of *C. reinhardtii* after 24 h exposure to NSAIDs at EC50 concentration. The growth rate was calculated using the formula K = [ln(N_1_) - ln(N_0_)]/t, where N_1_ is the number of cells after 1 day and N_0_ is the initial number of cells. Data are presented as averages ± SE. Different letters indicate significant differences at p ≤ 0.05 (ANOVA followed by Tukey's HSD test).

|  | **C** | **FFA** | **NBT** | **IBU** | **NPX** |
| --- | --- | --- | --- | --- | --- |
| **Growth rate [day^-1^]** | 3.13 ± 0.12a | 2.38 ± 0.04b | 2.45 ± 0.11b | 2.65 ± 0.04b | 2.66 ± 0.04b |

**Table S3.** Sequences of the primers for *Chlamydomonas reinhardtii* genes (according to <https://www.ncbi.nlm.nih.gov/genbank/>) analyzed in the control. and NSAIDs treated cultures.

| **Gene** | **Protein encoded** | **Primer sequence from 5′ end to 3′ end** |
| --- | --- | --- |
| *MSD3* | Isoform of manganese superoxide dismutase (Mn-SOD) | ACCAGACCCACCCAGGAG : CAGCCCCAACCAGGATAAC |
| *MSD5* | Isoform of manganese superoxide dismutase (_t_Mn-SOD) | CACAGCCAACCAGGACAACC : CGCGGACCAGTTTATCAG |
| *MSD4* | Isoform of manganese superoxide dismutase (Mn-SOD) | CCATGAGTGGGTTGTCCTG :  GACACTGGAACCACCAGATG |
| *FSD1* | Chloroplastic isoform of iron superoxide dismutase (Fe-SOD) | CCGTGGTGGAGGGCAAGACCCCCAT: TCGCCTGGCGCCGACATGCTTGTGA |
| *APX1* | Isoform of ascorbate peroxidase (APX) | TCAAGGAGATCAAGGCCAAG : GCCGCTCAGTCCAGAGTAAC |
| *APX2* | Isoform of ascorbate peroxidase (APX) | GTTCGCACCGTAGTCACAAA : GTGACAGGTCCCGTAGCTTG |
| *APX4* | Ascorbate peroxidase (APX 4) | AGGTGGCTGGGTTGATAACG : CAAAATCGGAGCACCTTGCC |
| *CAT1* | Mono-functional catalase (CAT 1) | CACGAGGGCTTCATGAACTT : CTTGGCGATCACCATCTTCT |
| *18S (rererence)* | 18S ribosomal RNA (18S) | GTAGTCATATGCTTGTCTC : GGCTGCTGGCACCAGACTTGC |

**Table S4.** *In silico* analysis of protein localization in *Chlamydomonas reinhardtii* with PredAlgo. Darker blue indicates the predicted location. Lighter blue indicates the location where p>0.5.

| **Gene** | **Signals** | **Membrane types** | **Cytoplasm** | **Nucleus** | **Extracellular** | **Cell membrane** | **Mitochondrion** | **Plastid** | **Endoplasmic reticulum** | **Lysosome/ Vacuole** | **Golgi apparatus** | **Peroxisome** |
| --- | --- | --- | --- | --- | --- | --- | --- | --- | --- | --- | --- | --- |
| ***APX1*** | Chloroplast transit peptide | Soluble | 0.1082 | 0.0864 | 0.0362 | 0.0274 | 0.1983 | 0.9247 | 0.0143 | 0.0217 | 0.0173 | 0.0017 |
| ***CAT1*** | Peroxisomal targeting signal | Soluble | 0.7118 | 0.2398 | 0.3313 | 0.4196 | 0.2591 | 0.0356 | 0.1272 | 0.1710 | 0.1446 | 0.6791 |
| ***FSD1*** | Chloroplast transit peptide | Peripheral Soluble | 0.0974 | 0.0715 | 0.0331 | 0.0368 | 0.1634 | 0.8936 | 0.0073 | 0.0240 | 0.0312 | 0.0039 |
| ***MSD4*** | Signal peptide | Soluble | 0.1259 | 0.0680 | 0.7527 | 0.1548 | 0.0318 | 0.1666 | 0.3267 | 0.3807 | 0.2426 | 0.0058 |
| ***MSD3*** | Signal peptide | Soluble | 0.1586 | 0.0322 | 0.8173 | 0.1480 | 0.0450 | 0.1069 | 0.2724 | 0.6811 | 0.3861 | 0.0020 |
| ***MSD5*** | Peroxisomal targeting signal | Soluble | 0.3775 | 0.0325 | 0.6425 | 0.1470 | 0.5835 | 0.4677 | 0.1942 | 0.5154 | 0.1729 | 0.5692 |

**Table S5.** The table summarizes the subcellular localization predictions of *Chlamydomonas reinhardtii* enzymes generated using various bioinformatics tools, including DeepLoc2.1 UniProt and PredAlgo.

| **Gene** | **deeploc2.1** | **UniProt** | **PredAlgo** |
| --- | --- | --- | --- |
| ***APX1*** | Plastid | - | Chloroplast |
| ***APX2*** | Plastid | - | - |
| ***APX4*** | Plastid | - | - |
| ***CAT1*** | Cytoplasm/peroxisome | Peroxisome | Cytoplasm/peroxisome |
| ***FSD1*** | Plastid | Chloroplast nucleoid | Chloroplast |
| ***MSD4*** | Extracellular | - | Secretory pathway |
| ***MSD3*** | Extracellular. Lysosome/Vacuole | Cytoplasm | Secretory pathway |
| ***MSD5*** | Extracellular/ Mitochondrion/ Peroxisome | - | - |

**Figure S2** . Effects of flufenamic acid (FFA), nabumetone (NBT), ibuprofen (IBU), and naproxen (NPX), applied at EC50 concentrations on protein content in *C. reinhardtii* cells. Data are presented as averages ± SE and expressed as a percentage of the control.

**Figure S3.** SOD isoenzymes PAGE analysis of *C. reinhardtii* cells treated for 24 h with (A) flufenamic acid (FFA), nabumetone (NBT), and (B) ibuprofen (IBU), naproxen (NPX). SOD- Cu/Zn-SOD reference pattern of defined activity 4.4 U·mg^-1^ protein (Sigma-Aldrich).

|  |  |
| --- | --- |

**Text S2.** Chlorophyll *a* fluorescence *in vivo* and photosynthetic pigments content.

Chlorophyll *a* fluorescence *in vivo* was measured using Handy Pea fluorometer with dedicated software PEA+ (Hansatech Ltd., UK), following Pokora et al. (2017). Before measurement, cell suspensions were adjusted to OD_680_= 0.2 ± 0.01, dark-adapted for 15 min, and measured at room temperature under a saturated light pulse (3000 μmol photons m^-2^ s^-1^). Photosynthetic pigments were determined spectrophotometrically after three extractions with 80% acetone (Majewska et al., 2021). Briefly, cell suspensions were centrifuged (2000 x g, 10 min, room temperature) and the pellets were disrupted with glass beads using a FastPrep-24 homogenizer (MP Biomedicals, USA). After clarification by centrifugation, the extracts were collected and analyzed in a 96-well plate at 447, 647, 664, and 740 nm using a Varioskan Flash Multimode Microplate Reader (Fisher Scientific, USA). Pigment concentrations were calculated according to Jeffrey & Humphrey (1975) and Lichtenthaler & Wellburn (1983), with path length correction according to Warren (2008).

**Table S6.** Chlorophyll *a* fluorescence parameters and photosynthetic pigments content in control, IBU- and NPX-treated cells of *C. reinhardtii* after 24 h exposure. Data are presented as average ± SE. Different letters indicate significant differences among groups (p ≤ 0.05; ANOVA or Kruskal-Wallis test followed by Tukey's HSD or Dunn’ s test).

| Parameters | C | IBU | NPX | Parameter description |
| --- | --- | --- | --- | --- |
| ABS/RC | 1.94 ± 0.04a | 4.38 ± 0.29b | 2.99 ± 0.12c | energy absorption per active reaction center |
| DI_0_/RC | 0.38 ± 0.02a | 2.12 ± 0.26b | 1.24 ± 0.10c | energy dissipation per active reaction center |
| TR_0_/RC | 1.55 ± 0.01a | 2.26 ± 0.10b | 1.73 ± 0.02c | energy trapping (Q_A_ reduction) per active reaction center |
| ET_0_/RC | 0.84 ± 0.00a | 0.86 ± 0.03a | 0.83 ± 0.01a | efficiency of electron transport per active reaction center |
| φP_0_ | 0.81 ± 0.01a | 0.53 ± 0.03b | 0.61 ± 0.02b | maximum quantum yield of primary photochemistry |
| ψE_0_ | 0.54 ± 0.01a | 0.38 ± 0.01b | 0.48 ± 0.01c | probability that a trapped exciton transfers an electron from Q_A_ to Q_B_ |
| φE_0_ | 0.44 ± 0.01a | 0.20 ± 0.01b | 0.30 ± 0.01b | the maximum quantum yield of electron transport further than Qa, |
| Chlorophyll *a* [pg/cell] | 0.59 ± 0.02a | 0.83 ± 0.05b | 0.77 ± 0.05b | chlorophyll *a* content |
| Chlorophyll *b* [pg/cell] | 0.39 ± 0.01a | 0.56 ± 0.03b | 0.48 ± 0.03b | chlorophyll *b* content |
| Carotenoids [pg/cell] | 0.159 ± 0.003a | 0.282 ± 0.012b | 0.253 ± 0.015b | carotenoids content |

Related data for FFA and NBT are available in our previous publication (Kapuścińska et. al., 2024).
